# Association of salivary alpha-2-macroglobulin with glycemia and glycated hemoglobin in type 2 diabetes mellitus: a systematic review and meta-analysis study

**DOI:** 10.1590/1516-3180.2021.0816.R2.19052022

**Published:** 2022-09-12

**Authors:** Douglas Carvalho Caixeta, Pedro Rogério Camargos Pennisi, Douglas Vieira Moura, Marjorie Adriane Costa Nunes, Foued Salmen Espindola, Cauane Blumenberg, Luiz Renato Paranhos, Robinson Sabino-Silva

**Affiliations:** I PhD. Collaborative Researcher, Innovation Center in Salivary Diagnostics and Nanotheranostics, Department of Physiology, Institute of Biomedical Sciences, Universidade Federal de Uberlândia (UFU), Uberlândia (MG), Brazil.; II Undergraduate Dentistry Student, School of Dentistry, Universidade Federal de Uberlândia (UFU), Uberlandia (MG), Brazil.; III Master’s Student, Innovation Center in Salivary Diagnostics and Nanotheranostics, Department of Physiology, Institute of Biomedical Sciences, Universidade Federal de Uberlândia (UFU), Uberlândia (MG), Brazil.; IV MSc. Doctoral Student, School of Dentistry, Universidade CEUMA, São Luiz (MA), Brazil.; V PhD. Professor, Institute of Biotechnology, Universidade Federal de Uberlândia (UFU), Uberlândia (MG), Brazil.; VI PhD. Collaborative Researcher, Postgraduate Program on Epidemiology, Universidade Federal University de Pelotas (UFPel), Pelotas (RS), Brazil.; VII PhD. Professor, Division of Preventive and Social Dentistry, School of Dentistry, Universidade Federal de Uberlândia (UFU), Uberlândia (MG), Brazil.; VIII PhD. Professor, Innovation Center in Salivary Diagnostics and Nanotheranostics, Department of Physiology, Institute of Biomedical Sciences, Universidade Federal de Uberlândia (UFU), Uberlândia (MG), Brazil.

**Keywords:** Saliva, Biomarkers, Diabetes mellitus, Blood glucose, Salivary proteins and peptides, HbA1C, Glycemia, Salivary protein

## Abstract

**BACKGROUND:**

Chronically elevated alpha-2-macroglobulin (A2MG) in the blood has been correlated with diabetes and the HbA1c profile; however, no systematic review has been conducted to evaluate the association of A2MG salivary levels and glycemia or HbA1c levels in diabetes mellitus type 2 (DM2) patients.

**OBJECTIVE:**

To evaluate whether A2MG salivary levels are related to the glycemia or HbA1c levels in DM2 patients.

**DESIGN AND SETTING:**

Systematic review developed at Universidade Federal de Uberlândia (UFU), Brazil.

**METHODS:**

Eight databases were used as research sources. The eligibility criteria included studies that reported data regarding mean salivary A2MG and the correlation between glycemia and/or HbA1c levels of DM2 subjects (uncontrolled and well-controlled) and non-diabetic subjects. The risk of bias of the studies selected was assessed using the Joanna Briggs Institute (JBI) critical appraisal tools for use in JBI systematic reviews. Pooled correlation coefficients were estimated using the Hunter-Schmidt method. Study estimates were weighted according to their sample size, and heterogeneity was calculated using the chi-square statistic.

**RESULTS:**

Four studies on DM2 patients were included in this systematic review after careful analysis of 1482 studies. Three studies compared A2MG with HbA1c and glycemia. Overall, the correlation between A2MG and HbA1c was strong (r = 0.838). In contrast, the correlation between A2MG and glycemia was low (r = 0.354).

**CONCLUSION:**

The strong association between HbA1C and salivary A2MG suggests that this salivary protein has the potential to be a surrogate for HbA1C, if corroboratory further evidence is obtained through large-scale studies.

## INTRODUCTION

Type 2 diabetes mellitus (DM2) is a metabolic disorder caused by a combination of decreased insulin secretion and decreased insulin sensitivity in peripheral tissues, primarily in the liver, muscles and adipose tissue as target organs.^1^ Currently, glycemia levels and glycated hemoglobin-A1c (HbA1c) are the gold-standard parameters for diagnosing and monitoring DM2. HbA1c is suitable for reflecting glycemic control from the previous 2-3 months, in accordance with the half-life of red blood cells.^2^

Different diagnostic tools, such as glycemia, HbA1C and the oral glucose tolerance test (OGTT), are used in the diagnosis of diabetes. According to the American Diabetes Association (ADA) guidelines, individuals with glycemia concentration ≥ 126 mg/dl, HbA1C level ≥ 6.5% or two-hour plasma glucose value after 75-gram OGTT ≥ 200 mg/dl are considered to be people with diabetes.^3^ The blood tests are invasive and painful^4^ and may lead to development of finger calluses, poor peripheral finger circulation and risk of infection.^4^

However, the classical HbA1c tests require several reagents with relatively high cost, and need some laboratory platforms.^5^ This reduces the availability of HbA1c tests in low and middle-income countries, despite their well-recognized capability for diabetes surveillance.^6^ Consequently, other types of biological samples for evaluating glycemic control, such as salivary biomarkers, might be an attractive alternative for early detection and monitoring of DM2.

The major salivary glands secrete saliva in response to the autonomic nervous system, which regulates the salivation process, including the flow and concentration of some salivary components such as α-amylase, which provides a reliable measurement of the sympathetic response.^7^ We previously showed that diabetes promotes changes in the autonomic activity of salivary glands, affecting both acinar and ductal cells, which are reflected in salivary composition.^8,9^

Human saliva contains a wide variety of proteins, including enzymes derived from salivary glands, blood, microorganisms and gingival crevicular fluid.^10^ In this context, saliva may contain potential biomarkers for DM2, which could be used as alternative non-invasive biofluids for diagnosing and monitoring DM2. Diabetes mellitus affects both salivary composition and salivary flow, due to microvascular alterations, neuropathies and hormonal imbalances.^11^ In this regard, both salivary sugars and glycosylated proteins have been found to be capable of distinguishing between hyperglycemic and normoglycemic conditions.^12^

Alpha-2-macroglobulin (A2MG) is a glycoprotein produced by the liver that can be present in human blood plasma, cerebral spinal ﬂuid and saliva fluid.^13^ The molecular structure of A2MG (720 kDa) consists of an assembly of four 180 kDa subunits into two disulﬁde-linked dimers, which form a noncovalent association that completes the tetrameric quaternary structure of the protein.^14^ A2MG is a glycoprotein capable of inhibiting a broad spectrum of proteases, and it also regulates the activity of cytokines, hormones, growth factors and other proteins.^15^ It can be stimulated by several factors, including by cytokines related to activation of the NF-kB, C/EBPb and C/EBPd pathways.^16^ Thus, patients with diabetes with positively regulated acute-phase proteins frequently express higher concentration of A2MG synthesis. Therefore, the clearance of tetrameric α2-macroglobulin-protease complexes is higher and, in compensation, there is enhanced synthesis of entire A2MG molecules, thus resulting in a net increase in the non-tetrameric circulating complex.^17^ Furthermore, the condition of proteinuria in patients with diabetes also can induces greater protein synthesis in the liver, thereby increasing the concentration and activity of plasma A2MG.^18^

Chronically elevated A2MG in the blood has been correlated with diabetes.^19,20^ Moreover, plasma A2MG levels have been correlated with the HbA1c profile.^21^ High serum A2MG levels could decrease the bioavailability of insulin and lead to impairment of blood sugar control.^4,22^ Salivary proteomic analysis on DM2 cases has indicated that A2MG was increased in subjects with uncontrolled diabetes, compared with prediabetic subjects.^23,24^ Furthermore, Aitken et al. (2015) and Chung et al. (2016) suggested that the level of salivary A2MG could be used as a surrogate for glycemic control in diabetic patients and that this protein represents a potential non-invasive alternative method for evaluating metabolic control.^22,25^ In this way, A2MG salivary levels could be useful as an alternative auxiliary tool for diagnosing DM2.

## OBJECTIVE

The aim of the present systematic review was to answer the following guiding question: “Are A2MG salivary levels related to glycemia or HbA1c levels in DM2 patients?” We tested the following hypothesis: salivary A2MG concentrations are correlated with HbA1c and glycemia levels in uncontrolled DM2 patients, compared with well-controlled DM2 patients or normoglycemic subjects.

## METHODS

### Protocol and registration

The protocol for this study was reported in accordance with the Preferred Reporting Items for Systematic Review and Meta-Analysis Protocols (PRISMA-P)^26^ and was submitted to the International Prospective Register of Systematic Reviews (PROSPERO) database, under the number CRD42020183831 (registration date: July 5, 2020), available from: https://www.crd.york.ac.uk/prospero/. This systematic review was reported following the guidelines for the Preferred Reporting Items of Systematic Review and Meta-Analysis (PRISMA)^27^ and was conducted in accordance with the Joanna Briggs Institute (JBI) Manual.^28^

### Eligibly and exclusion criteria of the study

Studies were included if they were observational studies (cross-sectional) among patients with uncontrolled type 2 diabetes mellitus and if they also assessed the correlation between salivary A2MG concentration and blood sugar level and/or serum HbA1c, compared with well-controlled DM2 patients or normoglycemic subjects. Studies were selected without restriction regarding their year and publication status (published or accepted/ahead of print articles).

The exclusion criteria consisted of the following situations: I) the study was unrelated to the objective; II) the study was a review article; III) the study was a follow-up or it assessed participants with other comorbid diseases, like patients with rheumatic diseases, terminal illnesses, chronic liver disease, chronic inflammatory processes in the oral cavity, chronic kidney disease in stages IV and V and autoimmune diseases; IV) the study did not report the procedures in accordance with the ethical standards.

### Sources of information and search

We searched for studies that evaluated salivary A2MG levels and serum glycemia and glycated hemoglobin (HbA1c) in type 2 diabetes mellitus cases. The MEDLINE (via PubMed), Scopus, LILACS, Web of Science, Embase and SciELO electronic databases were used as the primary study sources. In addition, OpenGrey and OpenThesis were used to partially capture the “gray literature”. MeSH (Medical Subject Headings), DeCS (Health Sciences Descriptors) and Emtree (Embase Subject Headings) were used to search the descriptors. The Boolean operators “and” and “or” were combined with the descriptors to improve the search strategy (Table 1 **)**. The bibliographic search was conducted up to a cutoff point of November 2020. In addition, we also manually checked the reference sections of the eligible studies and any indications by expert researchers, for the possibility of any additional studies that might have been missed by the electronic search. E-mails were sent out to three referral specialists for articles potentially eligible for this review.

**Table 1.** Strategies for database search

Database	Search strategy (November 2020)
PubMed (Best Match) http://www.ncbi.nlm.nih.gov/pubmed	((“Diabetes Mellitus Type 2” OR “Diabetes Mellitus, Noninsulin-Dependent” OR “Diabetes Mellitus, Non-Insulin-Dependent” OR “Diabetes Mellitus, Type II” OR “NIDDM” OR “Type 2 Diabetes” OR “DM2” OR “T2DM”) AND (“A2M protein, human” OR “α2-macroglobulin” OR “salivary α2-macroglobulin” OR “α2-MG” OR “alpha 2-macroglobulin” OR “A2MG”))
SCOPUS http://www.scopus.com/	(“Diabetes Mellitus Type 2” OR “Diabetes Mellitus, Noninsulin-Dependent” OR “Diabetes Mellitus, Non-Insulin-Dependent” OR “Diabetes Mellitus, Type II” OR “NIDDM” OR “Type 2 Diabetes” OR “DM2” OR “T2DM”) AND (“A2M protein, human” OR “α2-macroglobulin” OR “salivary α2-macroglobulin” OR “α2-MG” OR “alpha 2-macroglobulin” OR “A2MG”)
LILACS http://lilacs.bvsalud.org/	((“Diabetes Mellitus Type 2” OR “Diabetes Mellitus, Noninsulin-Dependent” OR “Diabetes Mellitus, Non-Insulin-Dependent” OR “Diabetes Mellitus, Type II” OR “NIDDM” OR “Type 2 Diabetes” OR “DM2” OR “T2DM”) AND (“A2M protein, human” OR “α2-macroglobulin” OR “salivary α2-macroglobulin” OR “α2-MG” OR “alpha 2-macroglobulin” OR “A2MG”))
Web of Science http://apps.webofknowledge.com/	((“Diabetes Mellitus Type 2” OR “Diabetes Mellitus, Noninsulin-Dependent” OR “Diabetes Mellitus, Non-Insulin-Dependent” OR “Diabetes Mellitus, Type II” OR “NIDDM” OR “Type 2 Diabetes” OR “DM2” OR “T2DM”) AND (“A2M protein, human” OR “α2-macroglobulin” OR “salivary α2-macroglobulin” OR “α2-MG” OR “alpha 2-macroglobulin” OR “A2MG”))
EMBASE https://www.embase.com	(‘diabetes mellitus type 2’/exp OR ‘diabetes mellitus type 2’ OR ‘diabetes mellitus, noninsulin-dependent’ OR ‘diabetes mellitus, non-insulin-dependent’/exp OR ‘diabetes mellitus, non-insulin-dependent’ OR ‘diabetes mellitus, type ii’/exp OR ‘diabetes mellitus, type ii’ OR ‘niddm’/exp OR ‘niddm’ OR ‘type 2 diabetes’/exp OR ‘type 2 diabetes’ OR ‘dm2’ OR ‘t2dm’/exp OR ‘t2dm’) AND (‘a2m protein, human’ OR ‘α2-macroglobulin’ OR ‘salivary α2-macroglobulin’ OR ‘α2-mg’ OR ‘alpha 2-macroglobulin’/exp OR ‘alpha 2-macroglobulin’ OR ‘a2mg’)
SciELO https://www.scielo.org/	((“diabetes mellitus type 2” OR “diabetes mellitus, noninsulin-dependent” OR “diabetes mellitus, non-insulin-dependent” OR “diabetes mellitus, type ii” OR “niddm” OR “type 2 diabetes” OR “dm2” OR “t2dm”) AND (“a2m protein, human” OR “α2-macroglobulin” OR “salivary α2-macroglobulin” OR “α2-mg” OR “alpha 2-macroglobulin” OR “a2mg”))
OpenGrey http://www.opengrey.eu/	“Diabetes Mellitus Type 2” OR “Diabetes Mellitus, Noninsulin-Dependent” OR “Diabetes Mellitus, Non-Insulin-Dependent” OR “Diabetes Mellitus, Type II” OR “NIDDM” OR “Type 2 Diabetes” OR “DM2” OR “T2DM” AND “A2M protein, human” OR “α2-macroglobulin” OR “salivary α2-macroglobulin” OR “α2-MG” OR “alpha 2-macroglobulin” OR “A2MG”
OpenThesis http://www.openthesis.org/	((“Diabetes Mellitus Type 2” OR “Diabetes Mellitus, Noninsulin-Dependent” OR “Diabetes Mellitus, Non-Insulin-Dependent” OR “Diabetes Mellitus, Type II” OR “NIDDM” OR “Type 2 Diabetes” OR “DM2” OR “T2DM”) AND (“A2M protein, human” OR “α2-macroglobulin” OR “salivary α2-macroglobulin” OR “α2-MG” OR “alpha 2-macroglobulin” OR “A2MG”))

### Study selection

Studies were selected in four stages. Initially, a calibration exercise was performed to fit pre-specified eligibility criteria and apply them to a small sample of the studies (20%) that had been retrieved, in order to determine inter-examiner agreement. After achieving an appropriate level of concordance (kappa ≥ 0.81), the reviewers (DCC and PRCP) performed a methodical analysis on all the study titles independently. Any disagreements between these examiners were discussed with a third reviewer (LRP), so as to reach a consensus.

In the first stage, the studies obtained from the databases were identified. The data were exported to the EndNote Web™ software (Thomson Reuters, Toronto, Canada), in which duplicates were removed. The remaining results were exported to Microsoft Word™ 2016 (Microsoft™, Redmond, Washington, United States), in which any remaining duplicates were manually removed.

In the second stage, all the titles were analyzed independently by the two reviewers, in order to determine their relevance. The reviewers were not blinded to the names of authors and journals. Titles that were not related to the topic were eliminated in this phase.

Then, in the third stage, the abstracts were reviewed in order to apply the exclusion criteria mentioned above. Titles in accordance with the aims of the present study but without abstracts available were fully analyzed in the fourth stage. In addition, expert investigators and potentially eligible studies found in the reference lists were included for subsequent analyses.

In the fourth stage, the full texts of the preliminarily eligible studies were obtained and evaluated to verify whether they did indeed fulfill the eligibility criteria, including expert investigators and potentially eligible studies found in the reference lists.

### Data collection

The two reviewers (DCC and PRCP) then independently accessed full-text copies of all eligible articles and collected data from each study using a pre-prepared spreadsheet. The following data were extracted from the studies: author, year, country, DM2 population, average age, average age range, gender ratio, diagnosis and collection period. In addition, information on the characteristics, preparation and measurement of the samples in the eligible studies was collected (saliva collection, saliva collection criteria, saliva preparation, blood collection, A2MG measurement, glycemia measurement and HbA1c measurement), along with the main results from the studies included (mean glycemia, mean HbA1c, mean A2MG, correlation of salivary A2MG with glycemia and correlation of salivary A2MG with HbA1c).

In order to ensure consistency between the reviewers, a calibration exercise was performed with both reviewers (DCC and PRCP), in which information was extracted jointly from an eligible study. Any disagreement between the reviewers was resolved through discussions, and if the disagreement continued, a third reviewer (LRP) was consulted to make a final decision.

### Risk of individual bias of the studies

The Joanna Briggs Institute Critical Appraisal Tools for use in JBI systematic reviews on observational (cross-sectional) studies^28^ were used to assess the risk of bias and the individual quality of the studies selected. Two authors (DCC and RSS) independently assessed each domain regarding its potential risk of bias, as recommended in the PRISMA statement.^27^

Each study was categorized according to the percentage of positive responses to the questions of the assessment tool. The risk of bias was considered high when 49% of the responses relating to the study in question were “yes” answers, moderate when 50% to 69% of the responses were “yes” and low when more than 70% of the responses were “yes”.^29^

### Statistical analyses

The correlations between the A2MG and DM2 biomarkers (glycemia or HbA1c) were considered in the meta-analysis. Correlation coefficients were pooled using the Hunter-Schmidt method^30,31^ and stratified according to the DM2 biomarker, for comparison with A2MG. Estimates using this method are weighted according to the sample size of each study. The correlation was considered perfect if the coefficients were equal to 1 or -1; strong if the coefficients ranged between |0.7| and |0.9|; moderate if the coefficients ranged between |0.4| and |0.6|; weak if the coefficients ranged between |0.1| and |0.3|; and zero if the coefficients were 0.^32^

The presence or absence of between-study heterogeneity was also assessed through the Hunter-Schmidt method using the chi-square statistic.^30,31^ The significance level was taken to be 5% in all analyses, which were all conducted using the Stata 16.1 software (StataCorp LLC, College Station, Texas, United States).

### Certainty of evidence

Quality of evidence and strength of recommendation were assessed using the Grading of Recommendation, Assessment, Development and Evaluation (GRADE) tool. The GRADE pro GDT software (http://gdt.guidelinedevelopment.org) was used for summarizing the results. This assessment was based on study design, methodological limitations, inconsistencies, indirect evidence, imprecision and other considerations. The quality of evidence was characterized as high, moderate, low or very low.^33^

## RESULTS

### Study selection

During the first phase of study selection, 1,581 results were found distributed in eight electronic databases, including the “gray literature”. After removing duplicate results, 1,482 articles remained for analysis of titles and abstracts.

In this phase, after a detailed analysis of titles and abstracts, only seven studies were found to be eligible for full-text analysis. The references of these seven potentially eligible studies were also carefully evaluated and one additional article was selected. Besides that, one article was indicated by an expert investigator, thus resulting in nine studies for full-text reading.

After reading the full text, five studies were found not to fulfil the inclusion criteria and were eliminated. Among these excluded studies, one^34^ was not related to the objective of this systematic review, two^23,24^ were proteomic analysis studies, one^35^ was a review study and another one^25^ was a follow-up study. Therefore, for these reasons, they were removed from further consideration.

**Figure 1.**
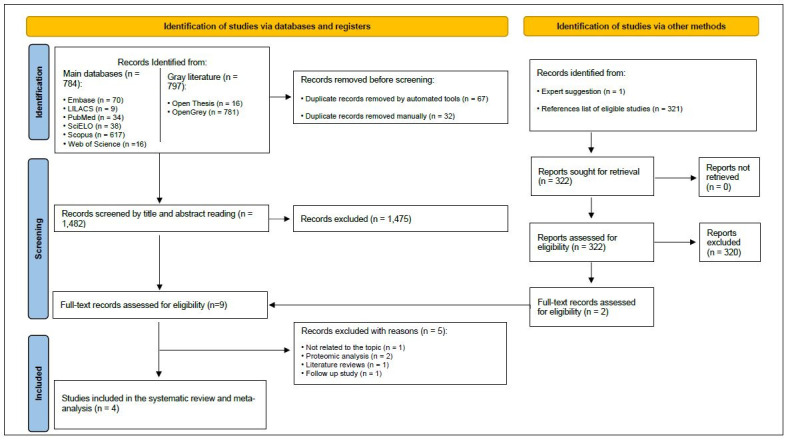
Flow-chart showing the search strategy, identification and inclusion/exclusion criteria used in the systematic review and meta-analysis.

Thus, four studies^22,36–38^ were selected for qualitative evaluation and meta-analysis. Figure 1 depicts the search, identification, inclusion and exclusion process for article selection.

### Study characteristics of eligible studies

The studies selected were published between 2015 and 2019 and were performed in Chile,^22^ China,^36^ Egypt^37^ and India.^38^ All studies^22,36–38^ had been approved by the ethics committee of their respective institution or hospital and also reported that informed consent had been obtained from the subjects prior to the start of the study. None of the articles used the STROBE checklist for cross-sectional studies.

Three studies included the sources of funding: Fondo Investigación Facultad de Odontología, Universidad de Chile (FIOUCH 13-002),^22^ ICMR Short Term Studentship Funding^38^ and nil (no funding).^37^ Other information regarding demographics and characteristics of the populations are presented in Table 2.

**Table 2.** Characteristics of the populations of the eligible studies included

Study	Country	Type of DM2 population	Control population	Average age (years)	Average age range (years)	Sex ratios	Diagnosis	Data collection period
Aitken et al.^22^	Chile	120 patients (75 patients with uncontrolled glycemia and 45 patients with well-controlled glycemia)	NA	61.6 ± 10.1	31-79	32.5% ♂, 67.5% ♀	Patients with HbA1c levels < 7% were classified as having adequate glycemic control and those with levels > 7% were classified as having inadequate glycemic control	July 2013 to December 2013
Feng et al.^34^	China	116 patients with DM2 and 60 patients with IFG (impaired fasting glucose)	60 healthy volunteers	DM2 (57 ± 12.3); IFG (55 ± 14.3); Control (51 ± 11.3)	Not reported	DM2 (54♂/62♀); IFG (27♂/33♀); Control (22♂/38♀)	American Diabetes Association in 2010 for DM2; IFG ≥ 7.0 mM (pre-diabetic); fasting blood glucose ranged from 5.6-6.9 mM (control)	February 2011 to March 2012
Nsr-Allah et al.^35^	Egypt	40 patients: 20 patients with uncontrolled glycemia (group 1) and 20 patients with well-controlled glycemia (group 2)	20 healthy volunteers (group 3)	Group 1 (49.75 ± 10.74); Group 2 (50.90 ± 10.54); Group 3 (48.9 ± 11.47)	23-65	Group 1 (7♂/13♀); Group 2 (9♂/11♀); Group 3 (13♂/7♀)	Patients with HbA1c levels < 7% were classified as having adequate glycemic control and those with levels ≥ 7% were classified as having inadequate glycemic control. Group 3 included with fasting plasma glucose less than 100 mg/dl and HbA1c less than 5.7%.	April 2016 and June 2017
Rastogi et al.^36^	India	87 patients: 53 patients with uncontrolled glycemia and 34 patients with well-controlled glycemia	NA	52.4 ± 8.1	35-65	43♂, 44♀	Not reported	August 2018 to October 2018

NA = not applicable; ♀ = women; ♂ = men; DM2 = type 2 diabetes mellitus; HbA1c = hemoglobin-A1c.

### Risk of bias within studies

All the studies presented a low risk of bias or high methodological quality. However, one study^38^ did not describe any specific information about the population and the parameters that assisted in making the diagnosis of diabetes. Therefore, this was indicated as unclear in the risk-of-bias table (Table 3).

**Table 3.** Risk of bias assessed using the Joanna Briggs Institute Critical Appraisal Tools for use in JBI Critical Appraisal Checklist for Analytical Cross-Sectional Studies^28^

Study	Q1	Q2	Q3	Q4	Q5	Q6	Q7	Q8	% Yes	Risk
Aitken et al.^22^	√	√	√	√	√	√	√	√	100	Low
Feng et al.^34^	√	√	√	√	√	√	√	√	100	Low
Nsr-Allah et al.^35^	√	√	√	√	√	√	√	√	100	Low
Rastogi et al.^36^	√	U	√	U	√	√	√	√	75	Low

Q1. Were the criteria for inclusion in the sample clearly defined?; Q2. Were the study subjects and the setting described in detail?; Q3. Was the exposure measured in a valid and reliable way?; Q4. Were objective, standard criteria used for measurement of the condition?; Q5. Were confounding factors identified?; Q6. Were strategies to deal with confounding factors stated?; Q7. Were the outcomes measured in a valid and reliable way? Q8. Was appropriate statistical analysis used? √ = yes; -- = no; NA = not applicable; U = unclear.

### Summary measurements and synthesis of results

Table 4 describes the correlation of salivary A2MG with glycemia and/or HbA1c and the respective means/standard deviations for glycemia, HbA1c and A2MG in the selected studies that were included in the quantitative analysis. All of these four studies were also included in the meta-analysis. However, only three studies compared A2MG with HbA1c,^22,36,37^ and only three studies compared A2MG with glycemia.^36–38^

**Table 4.** Summary of the main results from the studies included in the quantitative analysis.

Study	Mean glycemia	Mean HbA1c	Mean A2MG	Correlation of salivary A2MG with glycemia	Correlation of salivary A2MG with HbA1c
Aitken et al.^22^	NA	HbA1c > 7% (62.5%); HbA1c < 7% (37.5%)	Not reported	NA	r = 0.7748; P < 0.0001
Feng et al.^34^	DM2 (10.08 ± 2.44 mM); IFG (6.58 ± 0.24 mM); Control (5.01 ± 0.41 mM)	DM2 (8.7 ± 1.7%); IFG (5.8 ± 1.1%); Control (5.7 ± 0.7%)	Salivary A2MG (ng/ml): DM2 (192.6 ± 65.3); IFG (158.1 ± 60.1); Control (134.8 ± 63.2). Plasmatic A2MG (g/l): DM2 (1.70 ± 0.55); IFG (1.57 ± 0.36); Control (1.54 ± 0.38)	DM2 (r = 0.12, P = 0.199)	NA
Nsr-Allah et al.^35^	Group 1 (172.20 ± 26.52 mg/dl); Group 2 (100.65 ± 21.30 mg/dl); Group 3 (90.95 ± 8.66 mg/dl)	Group 1 (9.02 ± 1.38%); Group 2 (6.20 ± 0.61%); Group 3 (5.35 ± 0.44%)	Salivary A2MG (ng/ml): Group 1 (820.65 ± 190.17); Group 2 (331 ± 98.01); Group 3 (146.90 ± 42.01)	Group 1 (r = 0.586, P < 0.05); Group 2 (r = 0.146, P = 0.539); Group 3 (r = 0.650,P < 0.05); All subjects (r = 0.788, P < 0.001)	Group 1 (r = 0.778, P < 0.001); Group 2 (r = 0.666, P < 0.05); Group 3 (r = 0.474, P < 0.05); All subjects (r = 0.927, P < 0.001)
Rastogi, et al.^36^	Uncontrolled glycemia (290.58 ± 96.126 mg/dl); Well-controlled glycemia (172.83 ± 39.955 mg/dl)	HbA1c > 7% (60.9%); HbA1c < 7% (39%)	Salivary A2MG (ng/mL): Uncontrolled glycemia (2017.42 ± 575.133); Well-controlled glycemia (772.54 ± 118.324)	r = 0.660, P < 0.001	r = 0.977, P < 0.001

NA = not applicable; DM2 = type 2 diabetes mellitus; HbA1c = hemoglobin-A1c. A2MG = alpha 2-macroglobulin; IFG = impaired fasting glucose.

**Figure 2.**
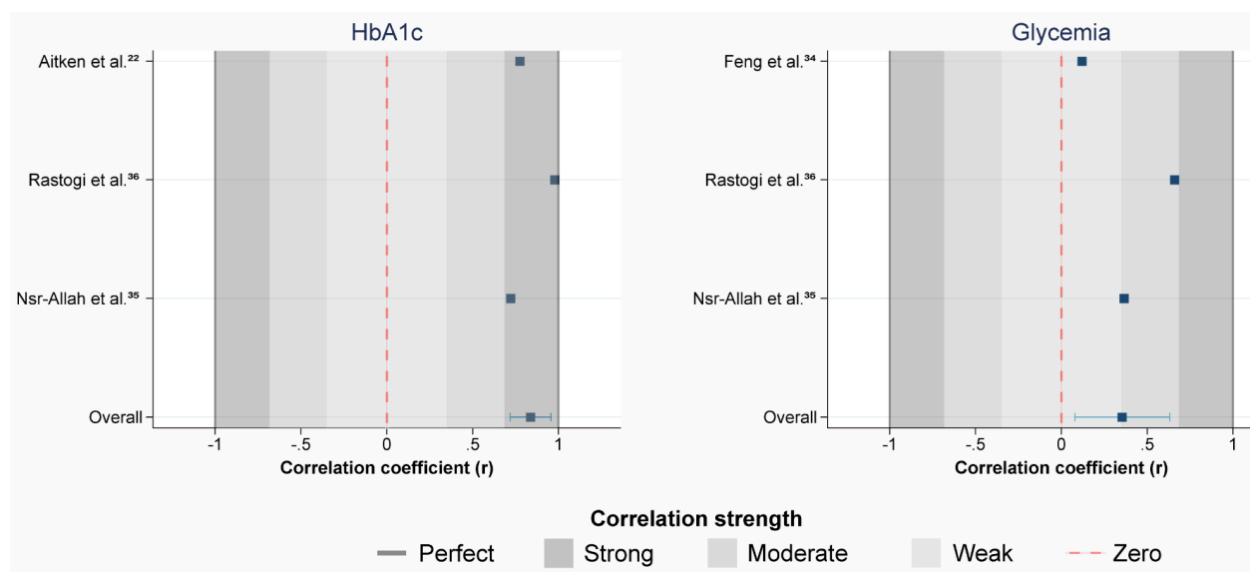
Correlations of salivary alpha 2-macroglobulin (A2MG) with hemoglobin-A1c (HbA1c) and glycemia.

The correlation between A2MG and HbA1c ranged from 0.722 to 0.977 in the three studies analyzed. Overall, the pooled correlation between these biomarkers was strong (r = 0.838; 95% confidence interval, CI: 0.719 to 0.956; P < 0.001) (Figure 2). In contrast, the pooled correlation between A2MG and glycemia was low (r = 0.354; 95% CI: 0.077 to 0.630; P = 0.006). Both meta-analyses presented significant heterogeneity between study results (P < 0.001); however, the heterogeneity levels were higher for glycemia analysis than for the HbA1c analysis.

### Certainty of evidence

The GRADE tool^33^ assessed two outcomes. Both outcomes (correlation between A2MG and HbA1c and correlation between A2MG and glycemia) were categorized as very low level of certainty, which means that the true effect is likely to be substantially different from the estimated effect. Table 5 shows more details regarding each outcome.

**Table 5.** Grading of Recommendations Assessment, Development, and Evaluation (GRADE) summary of findings table for the outcomes of the systematic review and meta-analysis

Quality assessment							Summary of results		
Number of studies	Study design	Risk of bias	Inconsistency	Indirectness	Imprecision	Publication biases	Number of participants	Effect	General quality
								r (95% CI)	
**Outcome 1: Correlation between A2MG and HbA1c**									
**3**	Cross-sectional studies	Not serious	Serious^1^	Not serious	Serious^2^	Not serious	247	0.838 (0.719- 0.956)	⨁ VERY LOW
**Outcome 2: Reduction of salivary creatinine after dialysis**									
**3**	Cross-sectional studies	Not serious	Not serious	Not serious	Serious²	Not serious	243	0.354 (0.077- 0.630)	⨁ VERY LOW

CI = confidence interval, A2MG = alpha-2-macroglobulin; HbA1c = hemoglobin-A1c. GRADE Working Group grades of evidence High certainty: We are very confident that the true effect lies close to that of the estimate of the effect. Moderate certainty: We are moderately confident in the effect estimate: The true effect is likely to be close to the estimate of the effect, but there is a possibility that it is substantially different. Low certainty: Our confidence in the effect estimate is limited: The true effect may be substantially different from the estimate of the effect. Very low certainty: We have very little confidence in the effect estimate: The true effect is likely to be substantially different from the estimate of effect ^1^The heterogeneity (I^2^) among the studies was high (> 75%); ²The number of participants included in the meta-analysis was too low.

## DISCUSSION

We conducted a systematic review to evaluate whether the increase in salivary A2MG concentration was correlated with HbA1c and glycemia levels in blood, in DM2 patients. We showed that there was a strong correlation between salivary A2MG and HbA1c, but with a low level of certainty. Hence, further studies are needed in order to determine the potential for application of A2MG in salivary platforms. However, the low association between A2MG and glycemia levels suggests that A2MG is not an accurate salivary protein that can act as a surrogate in glycemia tests.

Considering that glycemia reflects the blood glucose levels at the moment of the analysis, this test presents limitations with regard to reflecting glucose control over prolonged periods.^38^ The HbA1c test has been recommended as a means for assessing variations in glucose tolerance in type 2 diabetic patients, for long-term monitoring of diabetes.^6^ In addition, HbA1c tests can be performed at any time of the day without concerns about the fasting and it can indicate the average plasma glucose concentration over two to three months.^40,41^

However, the classical HbA1c test is performed in laboratory settings and only have limited use in point-of-care (POC) devices.^5^ This reduces the availability of HbA1c tests in low and middle-income countries.^6^ Moreover, several biological factors such as clinical conditions that alter erythropoiesis, glycation rate and erythrocyte destruction, and analytical interferences such as hyperbilirubinemia, carbamylated hemoglobin, certain medications and hemoglobin variants, affect the alteration cutoff values of the HbA1C test.^42^ Our findings from this meta-analysis confirm the hypothesis that A2MG presents a strong correlation with HbA1c test.

In this context, the higher correlation between salivary A2MG and HbA1C levels indicates that saliva is a promising alternative biofluid for diagnosing and monitoring diabetes. Among the advantages, saliva is simple and non-invasive to collect; it is convenient to store; and, compared with blood, it requires less handling during clinical procedures. Hence, further studies should be carried out in order to investigate the clinical applicability of salivary A2MG as a surrogate for HbA1C in diagnosing and monitoring DM2.

This systematic review had some limitations. The absence of a control group in some studies included^22,38^ could be considered a limitation, but their analysis on uncontrolled hyperglycemic subjects and subjects with type 2 diabetes presenting suboptimal control is also clinically relevant. In addition, the GRADE evaluation found that there were high levels of inconsistency and imprecision in the results obtained through the meta-analysis, which means that the evidence obtained was of very low level and that, possibly, the effect estimate found may differ from the real effect. Further studies with larger populations should be carried out in order to minimize imprecisions: these should include normoglycemic subjects, uncontrolled diabetic subjects and well-controlled diabetic subjects. Although HbA1c levels reflect the average blood glucose levels during approximately the previous 75 days, the mean duration of diabetes was not included in these studies.

On the other hand, lastly, the absence of systematic reviews and meta-analyses in this field gives added importance and timeliness to the meta-analysis of the present study. In the future, it will be important to define the predictive power of salivary A2MG for estimating HbA1c levels.

## CONCLUSION

The present study described a strong association between HbA1C and A2MG levels in saliva, in uncontrolled DM2 subjects, compared with well-controlled DM2 patients or normoglycemic subjects. On the other hand, the meta-analysis suggests that there was a very low correlation between glycemia and salivary A2MG. Further large-scale studies are needed in order to be able to recommend salivary A2MG levels as alternative surrogate for HbA1c. Nonetheless, the present study suggests that this has a potential role in providing a clinically valuable advance towards salivary monitoring of diabetes.
